# In Vitro and In Vivo Osteogenic Activity of Titanium Implants Coated by Pulsed Laser Deposition with a Thin Film of Fluoridated Hydroxyapatite

**DOI:** 10.3390/ijms19041127

**Published:** 2018-04-10

**Authors:** Luyuan Chen, Satoshi Komasa, Yoshiya Hashimoto, Shigeki Hontsu, Joji Okazaki

**Affiliations:** 1 Department of Removable Prosthodontics and Occlusion, Osaka Dental University, 8-1 Kuzuhahanazono-cho, Hirakata, Osaka 573-1121, Japan; komasa-s@cc.osaka-dent.ac.jp (S.K.); joji@cc.osaka-dent.ac.jp (J.O.); 2 Department of Biomaterials, Osaka Dental University, 8-1 Kuzuhahanazono-cho, Hirakata-shi, Osaka 573-1121, Japan; yoshiya@cc.osaka-dent.ac.jp; 3 Department of Biomedical Engineering, Faculty of Biology-Oriented Science and Technology, Kindai University, 930 Nishimitani, Kinokawa, Wakayama 649-6493, Japan; hontsu@waka.kindai.ac.jp

**Keywords:** implant, pulsed laser deposition, fluoridated hydroxyapatite, rat femur model, osteogenic activity

## Abstract

To enhance biocompatibility, osteogenesis, and osseointegration, we coated titanium implants, by krypton fluoride (KrF) pulsed laser deposition, with a thin film of fluoridated hydroxyapatite (FHA). Coating was confirmed by scanning electron microscopy (SEM) and scanning probe microscopy (SPM), while physicochemical properties were evaluated by attenuated reflectance Fourier transform infrared spectroscopy (ATR-FTIR). Calcium deposition, osteocalcin production, and expression of osteoblast genes were significantly higher in rat bone marrow mesenchymal stem cells seeded on FHA-coated titanium than in cells seeded on uncoated titanium. Implantation into rat femurs also showed that the FHA-coated material had superior osteoinductive and osseointegration activity in comparison with that of traditional implants, as assessed by microcomputed tomography and histology. Thus, titanium coated with FHA holds promise as a dental implant material.

## 1. Introduction

Teeth are crucial to mastication, phonation, and aesthetics and are an important component of the mouth–jaw system [1]. Accordingly, dentition defects or edentulous tissues cause many issues that directly affect quality of life. These defects are principally due to periodontitis, trauma, and excision of tumors. At present, fixed bridges and crowns, as well as implanted or removable partial and complete dentures, are widely used to restore occlusal relationships and other important functions of the mouth–jaw system [2,3].

Since the osseointegration theory was formulated, dental implantation has proven to be a reliable treatment and is now extensively used [4]. The American Academy of Implant Dentistry defines osseointegration as “contact established without interposition of nonbone tissue between normal remodeled bone and an implant, entailing sustained transfer and distribution of load from the implant to and within bone tissue.” Titanium (Ti) and its alloys have many suitable characteristics for use in implants, including biocompatibility, osseointegration, high resistance to wear and corrosion, and low immunogenicity [5,6]. However, conventional, unmodified Ti implants may not withstand heavy occlusion in the early period of implantation and require at least three months of stress-free healing [7]. Such long recovery times may not be acceptable to patients who wish to quickly regain aesthetic appearance and function and may also increase the risk of infection, other periodontal issues, and even implant failure. Therefore, accelerating osseointegration and healing has become a research priority.

Novel surface-coating treatments, including sol-gel, chemical vapor phase deposition, and pulsed laser deposition (PLD), may accelerate osseointegration [8,9,10]. Of these, pulsed laser deposition has emerged in the last 15–20 years as one of the most popular, efficient, and straightforward techniques to deposit a wide spectrum of materials, especially on targets with complex shapes, including implants. PLD is essentially a method to grow thin films, in which the high energy of particles ejected from the target by a laser plume significantly enhances the crystalline quality of the film [11]. A strong bond also forms between film and substrate, while the roughness and thickness of the film are controllable [12,13]. Previous research has proven that thin film developed by PLD does not break easily and offers favorable physical properties [14,15].

Over the past few years, depositing hydroxyapatite on Ti or its alloys has attracted much attention [16,17] because of increased biocompatibility and similarity to human hard tissues. However, hydroxyapatite also degrades quickly, limiting osseointegration. Hence, fluoridated hydroxyapatite (FHA), in which the hydroxyl group in hydroxyapatite is selectively substituted for fluorine [18], has emerged in recent years because it is more stable [19,20] but similarly biocompatible and osteogenic [21,22]. Of note, fluoride ions released from FHA also enhance cell attachment, proliferation, and differentiation [23,24].

In this study, we attempted to deposit thin FHA films on Ti implants by KrF PLD. The morphology and physicochemical properties of the film were assessed, along with osseointegration and osteogenic activity in vivo and in vitro.

## 2. Results

### 2.1. Materials Fabrication

**Figure 1:**
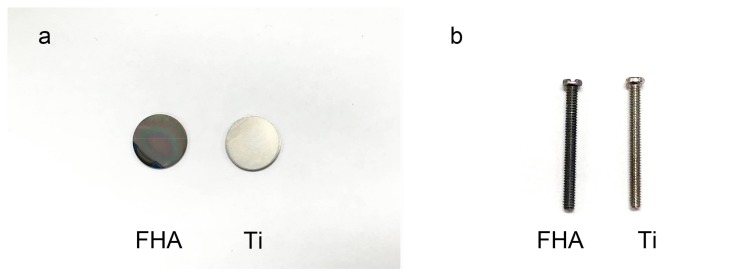
Gross appearance of Titanium (Ti) and fluoridated hydroxyapatite (FHA)-coated Ti (**a**) discs and (**b**) screws.

The gross appearance of uncoated Ti and FHA-coated materials are shown in Figure 1. Uncoated Ti discs appear light silver, while FHA discs are multi-colored. Regularly spaced ridges were observed in uncoated Ti discs in Figure 1a. Both FHA implant discs and screws were successfully coated, highlighting the effectiveness of PLD on targets of complex shape.

### 2.2. Surface Characterization

**Figure 2:**
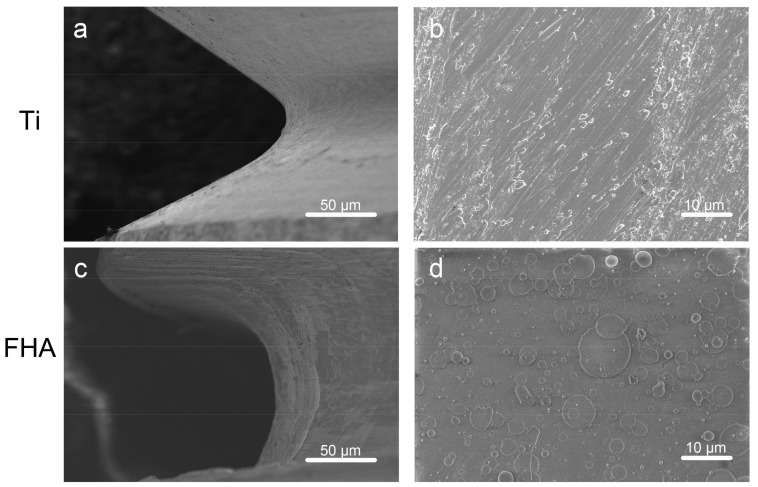
Scanning electron micrographs of (**a**,**b**) Ti and (**c**,**d**) FHA-coated screws.

**Figure 3:**
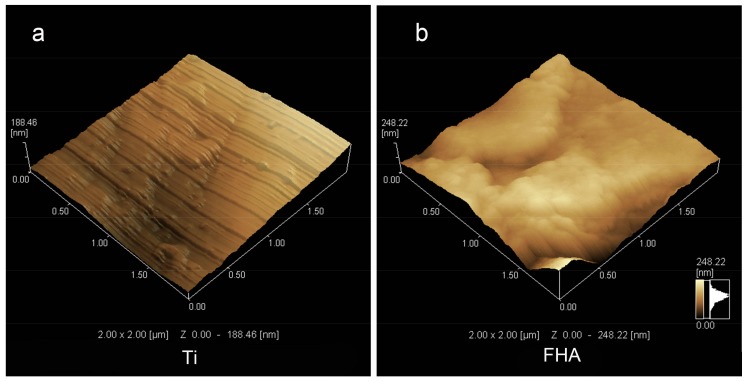
Scanning probe micrographs of (**a**) Ti and (**b**) FHA-coated surfaces.

**Figure 4:**
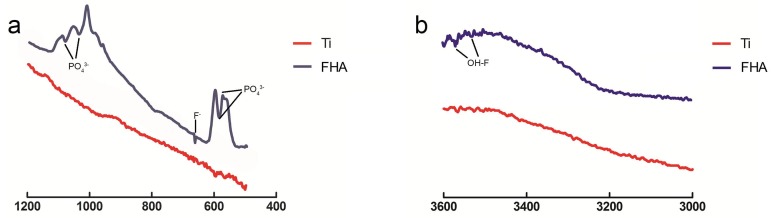
Fourier transform infrared spectra at (**a**) 1200–400 cm^−1^ and (**b**) 3600–3000 cm^−1^.

Scanning electron microscopy (Figure 2) confirmed that uncoated Ti screws contain regularly spaced ridges, and that FHA was successfully deposited. Meanwhile, no fracture due to brittleness was observed on the FHA implant surface. Scanning probe microscopy (Figure 3 and Table 1) indicated that surface roughness, measured as roughness values (Ra), was significantly higher for FHA-coated material (*p* < 0.05). With Fourier transform infrared spectroscopy (Figure 4), FHA-coated materials exhibited characteristic peaks of PO_4_^3−^ stretching vibration at 1090, 1040, 590, and 564 cm^−1^. OH–F stretching vibration with low frequency also appeared in FHA-coated devices, along with an F^−^ peak at 670 cm^−1^, indicating substitution of OH^−^ with F^−^.

### 2.3. Real-Time Quantitative Polymerase Chain Reaction (PCR)

**Figure 5:**
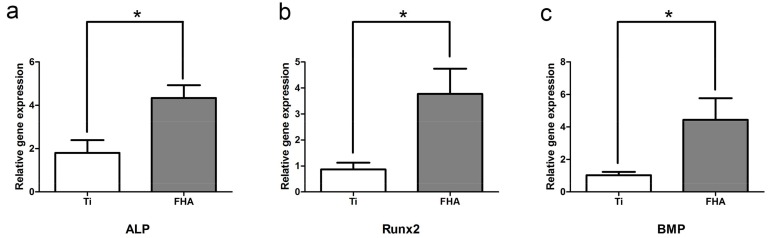
Expression of (**a**) ALP, (**b**) RUNX2, and (**c**) BMP in cells seeded on Ti and FHA-coated discs. * *p* < 0.05.

FHA-coated discs induced mRNA expression of alkaline phosphatase (ALP) and runt-related transcription factor 2 (RUNX2) within one week of seeding with rat bone marrow mesenchymal stem cells (Figure 5a,b); mRNA expression of bone morphogenetic protein (BMP) also increased at 21 days (Figure 5c) to levels significantly higher than on control Ti (*p* < 0.05).

### 2.4. Calcium Deposition in the Extracellular Matrix and Osteocalcin Production

**Figure 6:**
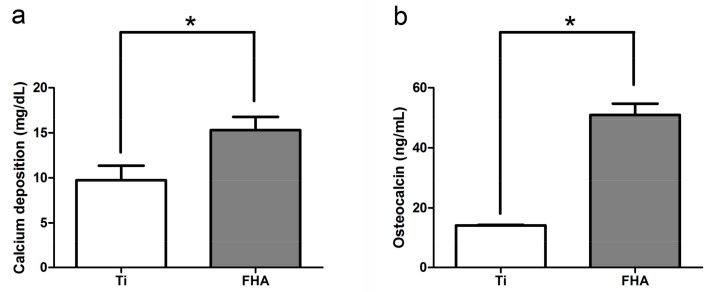
(**a**) Calcium deposition and (**b**) osteocalcin production in cells seeded on Ti and FHA-coated discs. * *p* < 0.05.

Calcium deposition, a marker of extracellular matrix mineralization, was higher at 28 days in the FHA-coated group than in the Ti group, as shown in Figure 6a (*p* < 0.05). Similarly, osteocalcin, a marker of late osteogenesis, was almost three times more abundant in the former than in the latter (*p* < 0.05).

### 2.5. Implantation into Rat Femurs

**Figure 7:**
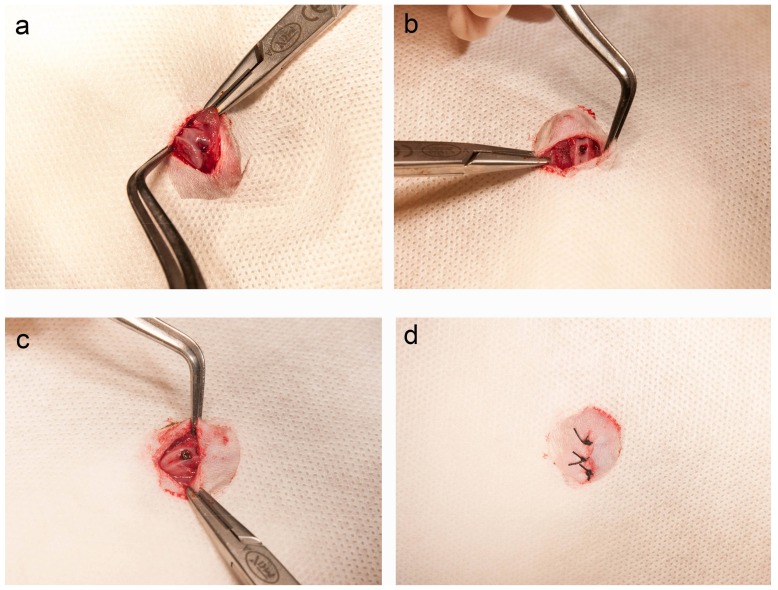
Implantation into rat femurs. (**a**) Incision; (**b**) Drilling of a hole; (**c**) Placement of implant; (**d**) Closure.

After vertical incision and exposure of the surgical field (Figure 7a), a circular hole was drilled using a dental bur into the intercondylar notch, with saline washing and with minimal bleeding (Figure 7b). A Ti screw was then securely and carefully implanted (Figure 7c). Finally, the excision was closed without tension (Figure 7d).

### 2.6. Microcomputed Tomography

**Figure 8:**
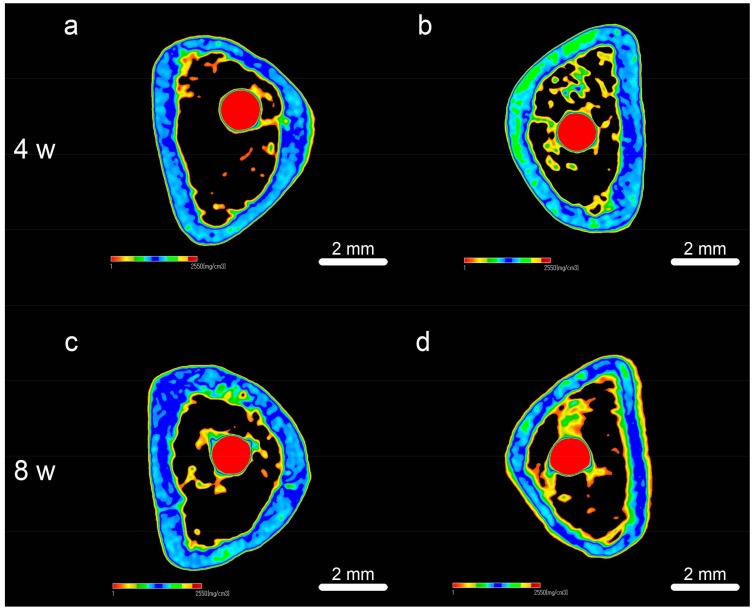
Transverse reconstructed microcomputed tomographs of (**a**,**c**) Ti and (**b**,**d**) FHA-coated implants after (**a**,**b**) 4 weeks and (**c**,**d**) 8 weeks.

**Figure 9:**
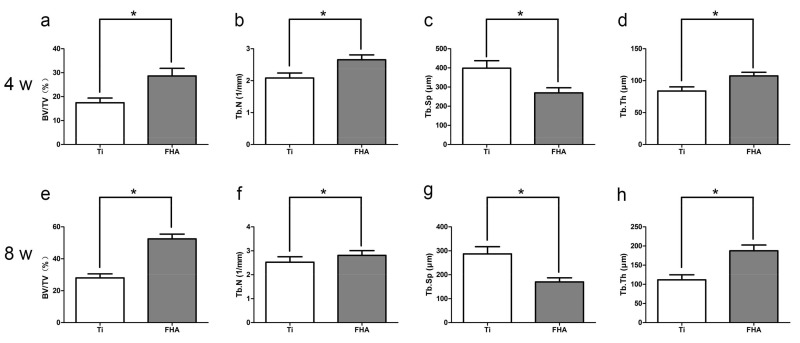
(**a**,**e**) Bone volume to total volume ratio (BV/TV), (**b**,**f**) mean trabecular number (Tb.N), (**c**,**g**) mean trabecular separation (Tb.Sp), and (**d**,**h**) mean trabecular thickness (Tb.Th) around implants after (**a**–**d**) 4 weeks (4 w) and (**e**–**h**) 8 weeks (8 w). * *p* < 0.05.

Reconstructed three-dimensional microcomputed tomographs of femurs with implants are shown in Figure 8, with cortical bone in green, cancellous bone in yellow, and implant in red. At both 4 weeks and 8 weeks, the ratio of bone volume to total volume (BV/TV), mean trabecular number (Tb.N), and mean trabecular thickness (Tb.Th) were significantly higher in FHA-coated implants, suggesting accelerated osteogenesis in the region of interest (Figure 9, *p* < 0.05). On the other hand, mean trabecular separation (Tb.Sp) was lower at both time points (*p* < 0.05).

### 2.7. Histology and Sequential Fluorescent Labeling

**Figure 10:**
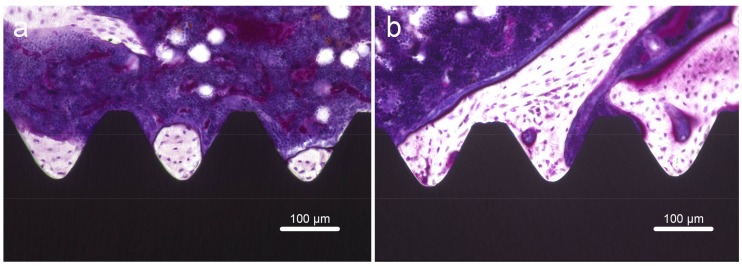
Villanueva staining of bone tissues around (**a**) Ti and (**b**) FHA-coated implants.

**Figure 11:**
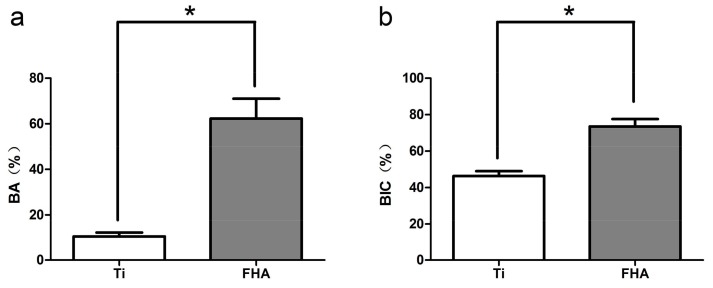
(**a**) Bone area ratio (BA) and (**b**) bone–implant contact (BIC) in Ti and FHA-coated implants. * *p* < 0.05.

Longitudinal sections were collected to assess formation of new bone around implants. Adverse inflammatory reactions or gaps at the bone–implant interface were not observed (Figure 10). At 8 weeks after surgery, the bone area ratio (BA) and bone–implant contact (BIC) were significantly higher around FHA-coated implants (Figure 11, *p* < 0.05). 

**Figure 12:**
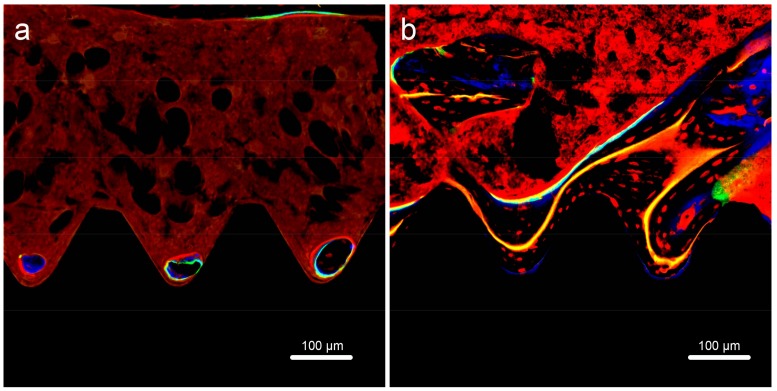
Fluorescent labeling of new bone and mineralization around (**a**) Ti and (**b**) FHA-coated implants.

**Figure 13:**
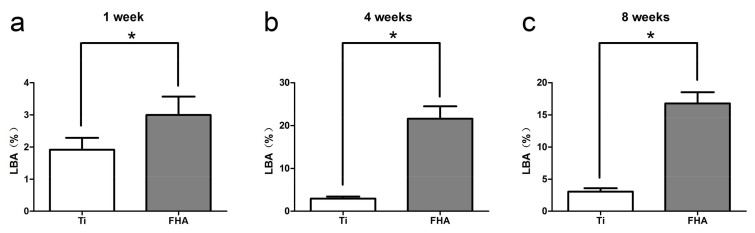
Fluorescently labeled bone area (LBA) after (**a**) 1 week, (**b**) 4 weeks, and (**c**) 8 weeks. * *p* < 0.05.

Bone formation around implants was also followed over time (Figure 12) by successive injection of oxytetracycline hydrochloride (blue) at 1 week, alizarin red S (red) at 4 weeks, and calcein (green) at 8 weeks. As assessed by confocal laser scanning microscopy, the labeled bone area between the implant surface and the boundaries labeled at 1 week, 4 weeks, and 8 weeks was significantly higher in FHA-coated implants (*p* < 0.05). Indeed, these implants were ossified with 1.5 times more new bone at 1 week compared with that of Ti implants and with more than four times more new bone at 4 and 8 weeks (Figure 13).

## 3. Discussion

A large number of patients worldwide require surgical implants to replace lost teeth [25]. However, functional restoration following implantation remains a clinical challenge. Hence, implants should exhibit great bone-bonding ability, excellent biocompatibility, and robust osteogenic activity. Materials that mimic bone, which mainly consists of calcium phosphate, are preferable for this purpose [26]. Such materials include hydroxyapatite, although it degrades quickly and is, thus, unsuitable for clinical use [27]. However, introducing fluoride ions may stabilize hydroxyapatite, as indicated by decreased solubility in simulated body fluids [28]. To this end, we prepared FHA by mixing hydroxyapatite and fluoroapatite at suitable concentrations.

We then coated Ti implants with FHA by KrF PLD. Visual inspection suggested that FHA was uniformly deposited as a thin film on both Ti discs and screws. Deposition was confirmed by SEM images of FHA screws, highlighting the effectiveness of PLD in coating metals or other materials with a thin film, as was previously observed with BaTiO_3_ and LiCoO_2_ thin film by PLD [29,30]. However, FHA thin film has better biocompatibility than that of the metallic oxide thin films mentioned because the biological features resemble those of human hard tissue. On the other hand, SPM indicated that coated surfaces were rough, with Ra value 24.48 vs. 5.83 for uncoated Ti surfaces, implying that the FHA-coated implant may promote cell attachment and growth because of its rough surface, likely by altering the amount and/or conformation of adsorbed proteins [31,32]. Indeed, surface properties, roughness, and composition are major determinants of the cellular response to implants [33]. Finally, an OH–F stretching peak with low frequency was observed with Fourier transform infrared spectroscopy, confirming the presence of fluoride ions in hydroxyapatite.

In early osteogenesis, the transcription factors ALP and Runx2 are abundantly expressed [34,35], while BMP is expressed in advanced stages [36]. Real-time PCR indicated that FHA-coated materials promote expression of these markers in the correct sequence, suggesting ordered and progressive bone formation. Indeed, FHA may degrade slowly and, hence, release calcium and fluoride ions chronically, of which the former may accelerate deposition of calcium salts, formation of calcium nodes and, thus, osteogenesis [37]. On the other hand, fluoride ions may not only prevent caries but also promote mineralization and crystallization of calcium phosphate during bone formation [38,39]. Therefore, both ions may support long-term osteogenesis. Accordingly, we found that calcium deposition in the extracellular matrix and cellular osteocalcin production were significantly higher in the FHA-coated group after 28 days, indicating that FHA promotes osteogenic differentiation not only at the genetic level but also at the cellular level. Similarly, Miao et al. [40] reported that FHA-coated Ti-6Al-4V significantly enhances the proliferation of osteoblast-like cells. These results imply that FHA retains the biocompatibility and osteogenic activity of hydroxyapatite [41,42] but has lower solubility, higher stability, and more clinical potential.

Consistent with in vitro data, FHA-coated screws implanted into rat femurs had better morphology and denser new bone in transverse view than that of uncoated Ti implants. Similarly, ossified areas in sagittal view were much larger around the former than around the latter. FHA coated implants were also more efficiently osseointegrated, as assessed by BIC [43]. There was no gap between implants and new bone tissue, which proved that after 8 weeks of implantation, FHA thin film could lead to good osseointegration and be substituted by new bone. We determined that this substitution process is similar to that of regenerative scaffold materials that are gradually substituted by new bone tissue [44,45]. In addition, time course analysis using fluorescent stains indicated sustained osteogenesis and osseointegration, perhaps as a result of chronic release of Ca^2+^, which not only promotes osteogenesis but also enhances blood clotting, a major driver of osseointegration, by activating inositol 1,4,5-trisphosphate receptors (IP3Rs), ryanodine receptors, and two-pore channels [46,47]. We note that although the rat femur does not completely replicate the intricate oral environment, it is used as a model of osseointegration and its consequences [48,49]. Nevertheless, further studies are required to assess the clinical value of FHA-coated implants, including in vivo degradation assays and oral implantation into larger animals.

## 4. Materials and Methods

### 4.1. Materials Fabrication

Commercially available grade 2 titanium discs (diameter 15 mm, thickness 1 mm) and screws (external diameter 1.2 mm, length 12 mm) were rinsed in an ultrasonic machine with acetone, ethanol, and deionized water, in this order. Devices were then coated by KrF PLD as described previously [50], using a mixture of hydroxyapatite and fluoroapatite (Ca_10_(PO_4_)_6_F_2_, Taihei Chemical Industrial, Osaka, Japan).

### 4.2. Surface Characterization

Surface topography was assessed on an S-4800 scanning electron microscope (Hitachi, Tokyo, Japan) operating at an accelerating voltage of 10 kV. Mean average surface roughness and two-dimensional surface topography were evaluated on an scanning probe microscope (Shimadzu, Tokyo, Japan). Physicochemical properties of Ti and FHA-coated Ti screws were analyzed by attenuated reflectance Fourier transform infrared spectroscopy over 400–4000 cm^−1^ on a Spectrum One instrument (Perkin Elmer, Norwalk, CT, USA).

### 4.3. Cell Culture

Experiments were performed under National Animal Care Guidelines (approval no. 16-08002, 2 August 2016). Femurs were isolated from two male rats aged 8 weeks, clipped at both ends, and flushed with media using a 21-gauge needle to collect bone marrow. Bone marrow mesenchymal stem cells were then cultured in 75-cm^2^ flasks according to a well-documented method [51]. Media were changed every three days.

### 4.4. Real-Time Quantitative PCR

Total RNA was extracted from cultured bone marrow mesenchymal stem cells after 7 and 21 days, using TRIzol^®^ Reagent according to the manufacturer’s protocol, and reverse transcribed using TaqMan reverse transcriptase-PCR (Life Technologies, Carlsbad, CA, USA). ALP and RUNX2 were quantified at 7 days by real-time quantitative PCR on a Step One™ Plus Real-Time RT-PCR system (Applied Biosystems, Thermo Fisher Scientific, Tokyo, Japan), using the 2^−∆∆*C*t^ method as previously described [52]. BMP was quantified in the same manner at 21 days.

### 4.5. Calcium Deposition in the Extracellular Matrix and Osteocalcin Production

To evaluate osteogenesis and mineralization of the bone extracellular matrix, rat bone marrow mesenchymal stem cells were seeded at 4 × 10^4^ /cm^2^ on Ti and FHA-coated Ti discs. After differentiation with 10 mM β-glycerophosphate, ascorbic acid, and 10 nM dexamethasone for 28 days, osteocalcin production and calcium deposition were evaluated using Rat Osteocalcin ELISA Kit (DS Pharma Biomedical Co., Ltd., Osaka, Japan) and Calcium E-Test Kit (Wako Pure Chemical Industries, Osaka, Japan), respectively, following the manufacturers’ instructions.

### 4.6. Implantation into Rat Femurs

This protocol was approved by Osaka Dental University Ethics Committee, Japan (approval no. 16-08002, 2 August 2016), and was compliant with National Animal Care Guidelines. Sixteen male Sprague-Dawley rats weighing 180–200 g and aged 8 weeks were randomly assigned to be implanted with Ti or FHA-coated Ti. After general anesthesia and surgical sterilization, a 10-mm vertical incision at the knee joint of the right hind limb was carefully made. The patella and joint tissue were then dislocated to expose the distal femur. Subsequently, a 1.2-mm hole in the intercondylar notch was drilled using a dental bur, with saline irrigation. Screws were then implanted, knee joints were reset, and incisions were sutured. Gentamicin (1 mg/kg) and buprenorphine (0.05 mg/kg) were injected for three days to prevent infection and reduce postsurgical pain.

### 4.7. Sequential Fluorescent Labeling and Microcomputed Tomography

To sequentially label new bone, animals were injected with 25 mg/kg oxytetracycline hydrochloride (Sigma, St. Louis, MO, USA) at 1 week after implantation, with 30 mg/kg alizarin red S (Sigma) at 4 weeks, and with 20 mg/kg calcein (Sigma) at 8 weeks. Rats were then anesthetized and sacrificed at either 4 or 8 weeks, and explanted femurs were imaged on an microcomputed tomography system (Shimadzu, Tokyo, Japan), with voltage 70 kV and current 118 μA. Three-dimensional reconstructions were obtained in Tri/3D-BON, and a cylindrical region of interest was set 2 mm below the highest point of the growth plate, extending 500 μm around implants. BV/TV, Tb.Th, Tb.N, and Tb.Sp were quantified to assess bone regeneration.

### 4.8. Histology of Sequentially Labeled Sections

After microcomputed tomography, femur specimens at 8 weeks were collected and stained by the Villanueva method to assess osseointegration and bone regeneration under a BZ-9000 digital cold illumination microscope (Keyence Co., Osaka, Japan) and an laser scanning microscope (Carl Zeiss, Oberkochen, Germany) [53]. The excitation and emission wavelengths were 351 and 460 nm for oxytetracycline hydrochloride, 543 and 617 nm for alizarin red S, and 488 nm and 517 nm for calcein. BA, BIC, and labeled bone area were measured using ImageJ in a 200× field around the implant and 2 mm below the growth plate.

### 4.9. Statistical Analysis

Groups were compared in SPSS 19.0 by Student’s *t* test and one-way analysis of variance, with *p* < 0.05 considered significant. Data are mean ± standard deviation.

## 5. Conclusions

We roughened smooth Ti implants by depositing a thin film of FHA using KrF PLD. These implants were found to promote osteogenesis in vitro and in vivo. In particular, FHA-coated implants elicited sustained osteogenesis and more efficient osseointegration in rat models than conventional uncoated Ti, as assessed by microcomputed tomography, histology, and sequential polychromatic fluorescent labeling. Therefore, FHA-coated devices have potential clinical value as dental implants.

## Figures and Tables

**Table 1:** Roughness values of Ti and FHA implant materials.

Device	Ra (nm)
Ti	5.83 ± 0.98
FHA	24.48 ± 2.94 *

* *p* < 0.05; Ra: roughness values.

## Abbreviations

KrF	krypton fluoride
FHA	fluoridated hydroxyapatite
Ti	titanium
PLD	pulsed laser deposition
SEM	scanning electron microscopy
SPM	scanning probe microscopy
ATR-FTIR	attenuated reflectance Fourier transform infrared spectroscopy
PCR	polymerase chain reaction
ALP	alkaline phosphatase
RUNX2	runt-related transcription factor 2
BMP	bone morphogenetic protein
BV/TV	bone volume to total volume
Tb.N	mean trabecular number
Tb.Th	mean trabecular thickness
Tb.Sp	mean trabecular separation
BA	bone area ratio
BIC	bone–implant contact
LBA	labeled bone area
